# Hydrocephalus treatment and outcome in African infants with myelomeningocele: what we have learned so far

**DOI:** 10.1186/1743-8454-7-S1-S3

**Published:** 2010-12-15

**Authors:** Benjamin C Warf

**Affiliations:** 1 Department of Neurosurgery, Harvard Medical School, Children's Hospital Boston, 300 Longwood Avenue, Boston, MA 02115, USA

## 

We estimate that around 2000 infants in Uganda develop hydrocephalus (HC) each year. Post-infectious hydrocephalus (PIH) accounts for 60% of all cases, and several lines of investigation are underway to determine the pathogens and their mode of transmission. HC associated with myelomeningocele (MM) is the second most common etiology, accounting for 15% of cases.

Access to treatment for hydrocephalus in Africa is inhibited by poverty, politics, poor infrastructure, and a paucity of neurosurgeons. These also present obstacles to follow up and emergency care for treatment failure, making death from future shunt malfunction a serious enduring threat.

We explored the role of endoscopic treatment for hydrocephalus in African infants, and found that combined endoscopic third ventriculostomy and bilateral choroid plexus cauterization (ETV/CPC) was significantly more successful than ETV alone in those less than one year of age. Infants with myelomeningocele benefitted the most from this approach, with a 76% success rate. In contrast to shunt failure, nearly all ETV/CPC failures become evident within 6 months of surgery. We have demonstrated factors that are independently predictive of ETV/CPC outcome, and have generated a new outcome prediction score (the Uganda Score) that is currently being evaluated across several centers in Africa.

In addition to safety and long-term efficacy of ETV/CPC in the myelomeningocele population, we also demonstrated that those treated in this way performed as well or better on the Bayley Scales of Infant Development over the course of their early childhood development than those who were shunted. Furthermore, we found no correlation between performance and ventricle size. We recently investigated whether the presence of HC or its method of treatment affected 5 year survival in these children, and were surprised to find no difference. Importantly, the most important determinant of survival was involvement in a community-based rehabilitation program. The five year mortality for these children was close to that of their unaffected peers (16%); whereas the mortality for those with no access to such a program was more than triple (50%). Deaths were mostly from causes not directly related to the underlying neurological conditions.

ETV/CPC is a safe and effective alternative to shunt-dependence in children with myelomeningocele. In Africa, any long-term survival advantage of shunt-independence may be obscured by diseases of poverty and neglect. “Life-saving surgery” in these children must be wedded to community-based support programs that promote their access to adequate health care and nutrition.

